# Visualization of charge carriers in photocatalysts

**DOI:** 10.1093/nsr/nwad188

**Published:** 2023-06-29

**Authors:** Si-Ming Wu, Markus Antonietti, Xiao-Yu Yang

**Affiliations:** State Key Laboratory of Advanced Technology for Materials Synthesis and Processing and Laoshan Laboratory, Wuhan University of Technology, China; Department of Colloid Chemistry, Max Planck Institute of Colloids and Interfaces, Germany; State Key Laboratory of Advanced Technology for Materials Synthesis and Processing and Laoshan Laboratory, Wuhan University of Technology, China

## Abstract

Surface photovoltage techniques combined with time-resolved spectroscopy methods provide an effective way to visualize the charge transfer dynamics in photocatalytic reactions.

Semiconductors can generate excited charge carriers for photo- or electrochemical reactions such as water splitting for H_2_ production. Now, the dynamic charge-transfer process has been directly observed to reveal the photocatalytic mechanism.

Illumination of a semiconductor with appropriate light causes an electron to transit from the valence to the conduction band and concurrent formation of charge carriers in the form of a positively charged hole and a higher-energy electron. As a result of this phenomenon, semiconductors are utilized as photocatalysts for redox reactions. For example, water can be split by such photocatalysts under solar illumination into elemental oxygen and hydrogen—a highly attractive energy-conversion and -storage cascade generally considered sustainable and clean [1]. Understanding the pathway(s) followed by the photogenerated carriers in the hydrogen and oxygen formation processes is crucial for designing photocells that make maximum use of charge carriers and produce hydrogen efficiently [2]. Although photocatalysts have been investigated for several decades, a detailed description of how photogenerated carriers are transferred on the surfaces/interfaces of semiconductors has stayed conceptual only. It is simply difficult to obtain meaningful information about the dynamic processes that take place at such short time and small size scales [3,4].

In an elegant study recently published in *Nature*, Li and co-workers devised unique methods involving time-resolved and spatiotemporally resolved measurements to monitor the charge-transfer processes taking place in photocatalytic reactions [4]. Their results disclosed more details of charge transfer in Cu_2_O semiconductors. Specifically, Li and co-workers showed that quasi-ballistic inter-facet electron transfer and spatially selective trapping are the predominant processes that facilitate efficient charge separation in photocatalysts. Knowledge gained about the mechanism, which comprises ultrafast-hot-electron-transfer and anisotropic-trapping regimes, provides in our opinion a new foundation for the rational design of high-performance semiconductors.

In conventional semiconductors, photogenerated charge transfer often occurs in a random manner and this disorder hinders the observation of dynamic photocatalytic process [5]. Li and co-workers exploited facet and defect engineering to produce a Cu_2_O semiconductor in which photogenerated electrons and holes accumulate separately on the respective {001} and {111} facets. Because of the anisotropic nature of the facets, charge transfer can be observed by using surface photovoltage microscopy (SPVM) [6]. Li and co-workers additionally employed time-resolved photoemission electron microscopy (TR-PEEM) to monitor the internal ultrafast charge transfer in the Cu_2_O lattice and transient surface photovoltage (SPV) analysis to follow surface-charge trapping (Fig. 1) [7]. Anisotropic charge transfer was demonstrated to be the critical factor causing improved performance of the new photocatalyst.

**Figure 1.**
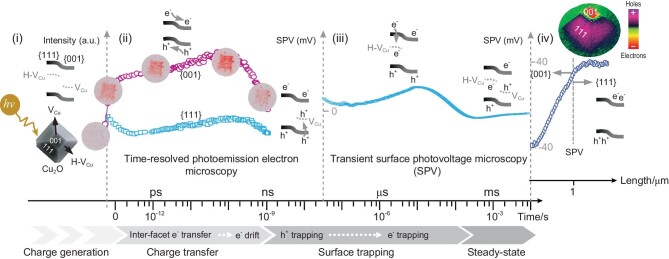
Schematic illustration of approaches used to monitor charge-transfer processes spatially and temporally in semiconductors, reproduced from [4]. (i) Morphology and band structure of CuO_2_ with spatially distributed defects on the {001} and {111} facets. (ii) The results of time-resolved photoemission electron microscopy (TR-PEEM) in the form of a plot of energy-integrated photoelectron intensity versus delay time, revealing that ultrafast charge generation, quasi-ballistic inter-facet e^−^ transfer and e^−^ drift take place on picosecond (10^−12^ s) to nanosecond (10^−9^ s) timescales. (iii) Transient SPV analysis that shows the occurrence of surface hole trapping on the {001} facet after a few nanoseconds and electron trapping on the {111} facet after tens of microseconds (10^−6^ s). (iv) SPV analysis displaying the localized charge distributions of electrons on {001} and holes on {111} facets.

Observations made in this investigation show that an understanding of charge-carrier transfer mechanisms, needed to create advanced semiconductor photocatalysts, arose from using very well-defined materials that have structural features that enable the use of key characterization techniques. The fundamental properties of a material, including particle size, morphology and defects, are critical factors governing not only the activity, but also the ability to decipher the mechanism of an operation of a photocatalyst. The electrodeposition method applied by Li *et al.* led to a form of Cu_2_O nanoparticles in which the sub-picosecond-scale accumulation of photogenerated electrons at the {001} facet permitted analysis by utilizing SPVM. Owing to the existence of high concentrations of copper vacancies, only electrons can be detected on the Cu_2_O surface using this technique. To change this contrast, Li and co-workers altered the deposition current density to produce Cu_2_O nanoparticles that have the significantly increased hydrogen densities required to compensate for copper vacancies. Due to their selective distribution on the respective {001} and {111} facets (Fig. 1i), the electrons and holes are then sufficiently spatially separated so that they can be detected using a combination of steady- and dynamic-state SPV techniques. It should be noted that the distance over which charges are transferred governs the ability to separate and observe the dynamics of the individual electron- and hole-transfer processes. It was found that Cu_2_O crystals with sizes of 4–6 μm allow the electrons and holes on the two facets to be clearly distinguishable.

Importantly, characterization techniques that provide spatial and temporal information were complementarily used to gain a detailed understanding of the dynamic processes occurring in materials [8]. In their study, Li and his co-workers used SPV to quantitatively assess the localized charge separation at Cu_2_O crystal surfaces, TR-PEEM to elucidate the nature of the ultrafast inter-facet electron transfer on the femtosecond (fs, 10^−15^ s) and nanosecond (ns, 10^−9^ s) timescales and finally transient SPV spectroscopy to analyse the charge-carrier trapping process at surface defects on the nanosecond-to-microsecond scale (Fig. 1ii–iv). The results reveal the holistic nature of the charge-transfer processes occurring in Cu_2_O and led the authors to propose a new quasi-ballistic transport mechanism as a replacement for the classical drift-diffusion model [9,10].

We believe that the surface photovoltage techniques for visualizing the separation and distribution of photogenerated charges in combination with time-resolved spectroscopy methods for elucidating the charge-transfer dynamics, which were utilized in this study, will serve as powerful tools for future investigations aimed at determining the mechanisms of processes promoted by other semiconductor-based photocatalysts. Going forward, applications of these approaches, potentially in combination with theoretical simulations, will allow the answers to other long-standing questions on mechanisms of virtually all photocatalysed reactions to be formulated and rational system engineering designs of high-performance photocatalytic devices to be crafted afterwards.
